# Systemic medications and dementia risk: an umbrella review

**DOI:** 10.1002/alz.085608

**Published:** 2025-01-09

**Authors:** Naaheed Mukadam, Clara Belessiotis, Ying Feng Yap, Shivangi Talwar, Andrea Bruun, Wenqianglong Li, Harry Ward, Pilar Letrondo, Madeleine Morelli‐Batters, Rongyu Lin, Michelle Eskinazi, Talen Wright, Joseph Hayes

**Affiliations:** ^1^ University College London, London United Kingdom; ^2^ University College London, London, Greater London United Kingdom; ^3^ Barts NHS Trust, London United Kingdom; ^4^ University College London Hospital NHS Trust, London United Kingdom; ^5^ Royal Free London NHS Foundation Trust, London United Kingdom

## Abstract

**Background:**

Dementia is a growing global public health challenge. Previous meta‐analyses have found that systemic medications may modulate dementia risk. We aimed to provide an overview of this evidence.

**Method:**

We conducted an umbrella review of meta‐analyses (PROSPERO CRD42021226307). MEDLINE, AMED, PsycINFO, and Embase were searched from inception to 25^th^ January 2023. Only peer‐reviewed meta‐analyses examining dementia risk and systemic medications in humans were included, with no restrictions on language. Studies examining cognitive decline were excluded. Participant details, study types, diagnostic criteria, publication bias, heterogeneity, and summary effect estimates were extracted from publications. Study quality was assessed using the AMSTAR‐2. Data extraction and quality ratings were carried out independently by two authors.

**Result:**

60 studies were included in the final review. Included meta‐analyses examined anti‐hypertensives (n = 17), statins (n = 7), antacids (n = 7), non‐steroidal anti‐inflammatory drugs (NSAIDs) (n = 6), diabetes medications (n = 4), hormone replacement therapy (n = 4), androgen deprivation therapy (ADT) (n = 4), psychotropic medications (n = 4), anticholinergics (n = 3), anticoagulants (n = 2), and vitamin E (n = 2). Number of participants in each review ranged from 842 to 9,162,509. Mean age ranged from 59 to 78 years, and between 0 and 100% of participants were female. There was a trend towards decreased risk of dementia with antihypertensives, statins, NSAIDs, anticoagulants, and vitamin E, and towards increased risk with ADT, benzodiazepines, and anticholinergic medications.

**Conclusion:**

Available data were primarily observational, leading to biases such as confounding by indication, and few studies met a high number of critical quality criteria. Randomised‐controlled data were rare but supported an association between treatment of hypertension and reduced dementia incidence and a possible association between hormone replacement therapy and increased risk of dementia. Our review provides an overview of what kinds of medication are associated with dementia risk, to guide clinical practice and future research. Currently there are no systemic medications that should be prescribed with the primary aim of reducing dementia risk.

